# Optimal wavelength selection for optical spectroscopy of hemoglobin and water within a simulated light-scattering tissue

**DOI:** 10.1117/1.JBO.23.4.041202

**Published:** 2018-01-26

**Authors:** Mikael Marois, Steven L. Jacques, Keith D. Paulsen

**Affiliations:** a Dartmouth College, Thayer School of Engineering, Hanover, New Hampshire, United States; b Tufts University, Department of Biomedical Engineering, Medford, Massachusetts, United States

**Keywords:** reflectance, spectral fitting, wavelength selection

## Abstract

An algorithm that selects optimal wavelengths for spectral fitting of diffuse light reflectance spectra using a nonnegative least squares method is presented. Oxyhemoglobin, deoxyhemoglobin, and water are considered representative absorbers, but the approach is not constrained or limited by absorber selection provided native basis spectra are available. The method removes wavelengths iteratively from a scattering-modulated absorption matrix by maximizing the product of its singular values and offers considerable improvements over previously published wavelength selection schemes. Resulting wavelength selections are valid for a broad range of optical properties and yield lower RMS errors than other wavelength combinations. The method is easily modified and broadly applicable to tissue optical spectroscopy. Adaptation of the algorithm to select optimal light-emitting diodes for fitting blood is described.

## 1 Introduction

Multispectral reflectance is a broadly established method for estimating concentrations of multiple absorbers in tissue. It is often used to decouple and estimate quantities of oxyhemoglobin (${\mathrm{HbO}}_{2}$), deoxyhemoglobin (HbR), and water ($H_{2}O$), among other absorbers, through spectral fitting based on least squares, provided the acquisition of multiple wavelengths is available (a minimum of three wavelengths is needed for ${\mathrm{HbO}}_{2}$, HbR, and $H_{2}O$). The wavelengths acquired have a considerable effect on the accuracy of the concentration estimates that are generated. In the approach presented here, wavelengths are selected by maximizing the product of the singular values of the scattering-modulated absorption matrix. Analysis shows that this selection method is robust and stable over a wide range of optical properties and that it improves the accuracy of the resulting estimates relative to algorithms previously published. Finally, a practical application where optimal light-emitting diodes (LEDs) are chosen to estimate blood concentrations is described.

## 2 Methods

### 2.1 Spectral Fitting

Concentrations of multiple absorbers in tissue can be estimated from its wavelength-dependent reflectance signal. For each wavelength, the fraction of reflectance collected can be modeled with Beer’s law

$$R(\lambda )=\exp[-\mu_{a}(\lambda )L(\lambda )],$$ (1)

where $\mu_{a}(\lambda )$ is the wavelength-dependent absorption coefficient represented in the medium as a linear combination of the absorption coefficients of all components present. To illustrate the benefits of our approach, we consider oxyhemoglobin, deoxyhemoglobin, and water, and, accordingly, the absorption coefficient is

$$\mu_{a}(\lambda )=BS\mu_{\mathrm{oxy}}(\lambda )+B(1-S)\mu_{\mathrm{deoxy}}(\lambda )+W\mu_{\text{water}}(\lambda ),$$ (2)

where L(λ) is the average path length of photons in the medium at wavelength λ, which varies according to the absorption ($\mu_{a}$) and scattering ($\mu_{s}^{\prime }$) coefficients of the tissue, and is computed using an adaption of the diffusion model described by Farrell et al.^1^ B, S, and W quantify the blood concentration, oxygen saturation, and water concentration, respectively. Parameter estimation can be refined through more advanced models. For example, adding specific layers of tissue can be advantageous if the laminar distribution of chromophores is known (e.g., in the case of epidermial melanin). Additionally, correction factors accounting for vessel size can be added depending on the underlying structures that are being considered (notably, blue light does not penetrate to the center of large vessels and, therefore, underestimates blood content). The optical depth is defined as the natural logarithm of the reflectance signal and is needed to extract information about concentration through spectral fitting

$$-\ln\;R(\lambda )=\mu_{a}(\lambda )L(\lambda ).$$ (3)

Given an optical depth equation for each wavelength, the problem is decomposed into a matrix system

[−ln[R(λ1)]−ln[R(λ2)]⋮−ln[R(λN)]]⏟OD=[μa,oxy(λ1)L(λ1)μa,deoxy(λ1)L(λ1)μa,water(λ1)L(λ1)μa,oxy(λ2)L(λ2)μa,deoxy(λ2)L(λ2)μa,water(λ2)L(λ2)⋮⋮⋮μa,oxy(λN)L(λN)μa,deoxy(λN)L(λN)μa,water(λN)L(λN)]⏟μaL[BSB(1−S)W]⏟C, (4)

where OD is a 1×N vector of optical depths, $\mu_{a}L$ is a N×3 matrix of pathlength-modulated absorption coefficients associated with the absorbers of interest, and C is the concentration vector. The concentration vector can be computed using the Moore–Penrose pseudoinverse.^2^ Positivity constraints are added to ensure that no negative concentrations will be computed,^3^ yielding the least square fit

C=[(μaL)T(μaL)]−1[μaL]T⏟ODMoore−Penrose pseudoinverse. (5)

In principle, any three wavelengths can be used to generate concentration estimates, but their selection affects the resulting accuracy dramatically.^4^^–^^10^

### 2.2 Wavelength Selection

In this paper, optimal wavelengths are selected from an oversampled set of wavelengths and iteratively removed until the desired (minimum) number of wavelengths is found, based on a selection algorithm used by Luke et al.^4^^,^^5^ For each row (i.e., wavelength) being removed, selection criteria are computed to evaluate the importance of that wavelength. For clarity, a pseudocode for the selection algorithm is outlined here:

1. Start with matrix $\mu_{a}L$ that has an oversampled number of wavelengths.
2. Remove the first row (i.e., wavelength of matrix $\mu_{a}L$). Compute the value of the selection metric for this reduced matrix.
3. Iteratively repeat step 2 for all rows of $\mu_{a}L$.
4. Eliminate the row whose removal yielded the largest/smallest selection metric (depending on the metric).
5. Repeat these steps until the desired number of wavelengths is achieved.

Selection of optimal wavelengths is achieved by maximizing or minimizing a selection metric. This metric is often based on the singular values of the system’s absorption matrix (in this case, $\mu_{a}L$). These singular values indicate the orthogonality of the spectra being fitted and can be found through singular value decomposition. Previous studies have investigated the selection of optimal wavelengths for a wide variety of spectroscopic applications, including photoacoustic imaging,^4^^,^^5^ spatial frequency-domain imaging,^6^ and optical tomography.^8^^,^^9^ Most of these reports base selection on the matrix condition number (ratio of the largest to the smallest singular value).^4^^,^^6^^,^^8^ Luke and Emelianov^5^ also maximized the smallest singular value, which prevents loss in rank, and reported improved results using this approach. In this paper, we maximized the product of all singular values, which yielded concentration estimates that were more accurate than those generated from wavelengths selected by previously published algorithms. While the condition number indicates the spread in singular values and using the smallest singular values prevents the system from losing rank, both approaches discard information contained in the rest of the singular values whereas the product of singular values senses information from the full matrix spectrum.

To evaluate the performance of sets of optimal wavelengths generated by various methods, simulated spectra were generated from randomly chosen optical properties and concentrations of ${\mathrm{HbO}}_{2}$, HbR, and $H_{2}O$ from ranges reported in Table 1. Ten percent (10%) white Gaussian noise was added to each of these simulated spectra, and least square fits were performed to estimate concentrations. Root mean squared (RMS) errors between the estimates and the true concentrations were recorded. This process was repeated $10^{3}$ times for 10 randomly selected optical properties, and the resulting averaged RMS error was used to compare algorithms and evaluate the quality of selected wavelengths.

**Table 1:** Ranges of the parameters used to generate random absorption spectra.

Parameter	B	S	W	a	b
Range	0.001 to 0.003	0.5 to 0.95	0.65 to 0.8	0.33 to 3	0 to 3

Note: B is the blood volume fraction, S is the average arteriovenous oxygen saturation of hemoglobin, W is water volume fraction, and a and b specify the reduced scattering coefficient $\mu_{s}^{\prime }=a{\left(\lambda /500\;\mathrm{nm}\right)}^{-b}$, such that $a=\mu_{s}^{\prime }(500\;\mathrm{nm})$

## 3 Results

### 3.1 Selection Criterion

**Fig. 1:**
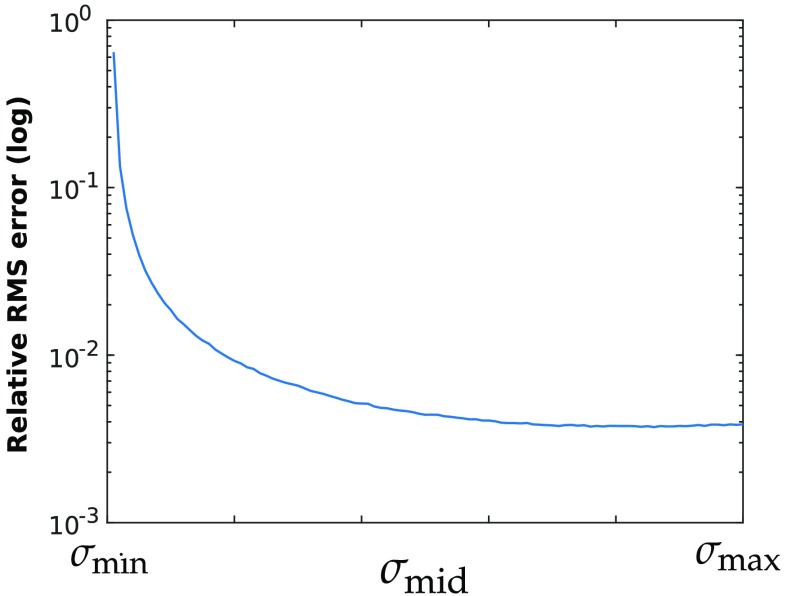
Average relative RMS error generated when performing spectral fitting for ${\mathrm{HbO}}_{2}$, HbR, and $H_{2}O$ with a center singular value varying from the smallest to largest singular value of the system.

To demonstrate that the center singular value of $\mu_{a}L$ has an important impact on the quality of the resulting least square estimates, simulated spectra of random optical properties were generated to populate $\mu_{a}L$ matrices, and their center singular values were artificially increased or decreased within the range of their extreme singular values. Least square fitting was performed with the scaled center singular value, and RMS errors were computed and are shown in Fig. 1.

**Fig. 2:**
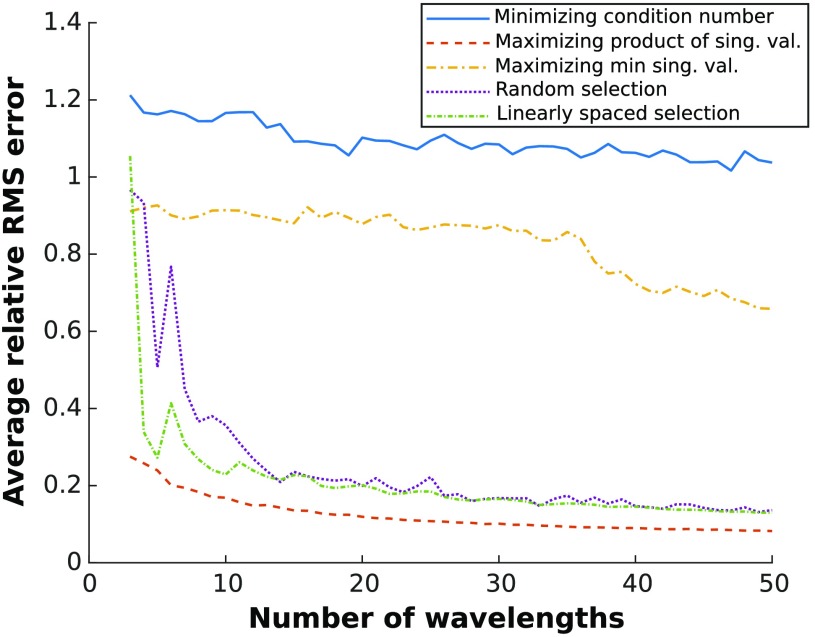
Average relative RMS error of concentration estimates generated by fitting ${\mathrm{HbO}}_{2}$, HbR, and $H_{2}O$ for wavelengths chosen using different selection methods. RMS error was averaged over 100 spectra of random optical properties contained in Table 1 ranges. The methods compared are: (1) minimizing the condition number of $\mu_{a}L$, (2) maximizing the product of the singular values, (3) maximizing the minimum singular value,^5^ (4) a random choice of wavelengths, and (5) linear spacing of wavelengths.

The error decrease shows that the size of the center singular value has a considerable effect on the accuracy of the concentration estimates. Artificially modifying the center singular value maintained both the condition number and smallest singular value of the system as constants whereas the average RMS errors varied over orders of magnitude. Accordingly, maximizing the product of singular values when selecting optimal wavelengths is a simple but desirable selection criterion. To further illustrate the advantage of this selection metric, average RMS errors generated when fitting with wavelengths chosen by different selection criteria were compared in Fig. 2. As a control, the RMS error associated with wavelengths selected randomly and linearly within the 480- to 1000-nm range was also computed.

These results demonstrate that using the product of the singular values yields improved results over condition number or smallest singular value, especially when selecting a larger number of wavelengths. Interestingly, random and linearly spaced wavelength selection outperformed those based on condition number and the smallest singular value. Methods that rely on the smallest singular values are affected by spectra that are highly disproportioned (as is the case here, with water having an absorption coefficient that is orders of magnitude lower than blood).

### 3.2 Optimal Wavelengths

**Fig. 3:**
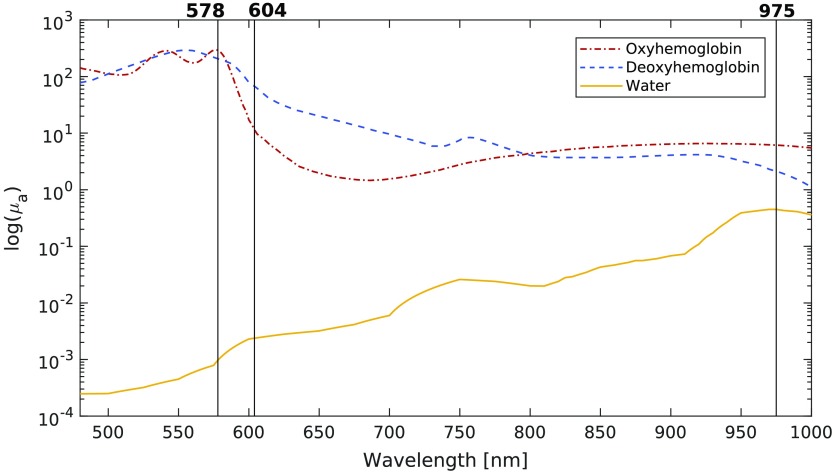
Optimal wavelengths selected by maximizing the product of the singular values of the system.

Using our selection algorithm based on maximizing the product of singular values, the three optimal wavelengths for estimating blood concentration and saturation in tissue were computed for a wide variety of optical properties. For the majority (76%) of optical property combinations tested, the same trio of optimal wavelengths was selected. These wavelengths are shown in Fig. 3.

**Fig. 4:**
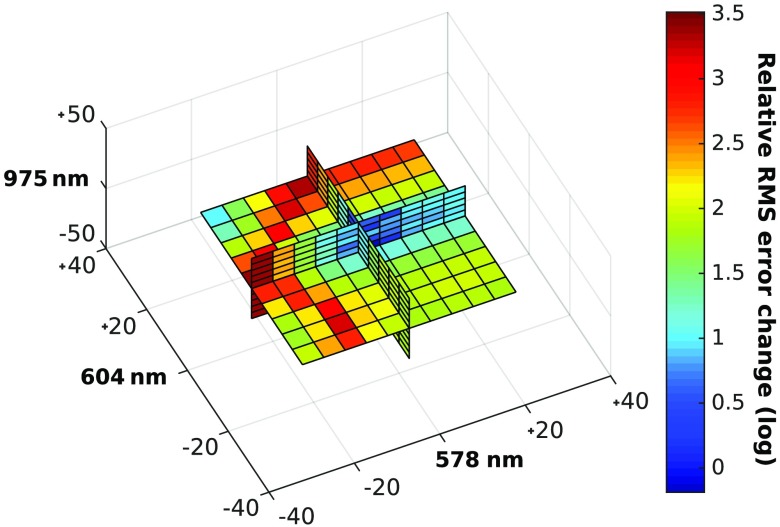
Relative change in average RMS error for wavelength trios around the optimal wavelengths selected by maximizing the product of the singular values. Each square represents a 5-nm variation.

In the other cases, which were mostly when oxygen saturation was lower than 60%, the center wavelength selected switched from 604 to 596 nm. Variations in the wavelengths picked are expected when modifying optical properties since they alter the shape of the absorption spectra used to compute the optimal wavelengths. Even if, for some combinations of optical properties, the selection algorithm found wavelengths that differed slightly from the ones in Fig. 3, these choices generated similar RMS errors. The concentration estimates generated with the set of optimal wavelengths highlighted in Fig. 3 yielded RMS errors that were smaller than the ones generated with other wavelength trios, on average. The region of low RMS error was constant for a radius of about 10 nm around the optimal wavelengths, as shown in Fig. 4, in which the change in RMS error relative to the error generated with the optimal wavelengths is presented.

Interestingly, some wavelengths can be moved with greater freedom than others. For example, increasing the 578-nm wavelength changes RMS error nominally, while decreasing it produces RMS errors that are orders of magnitude greater.

### 3.3 Additional Chromophores

**Fig. 5:**
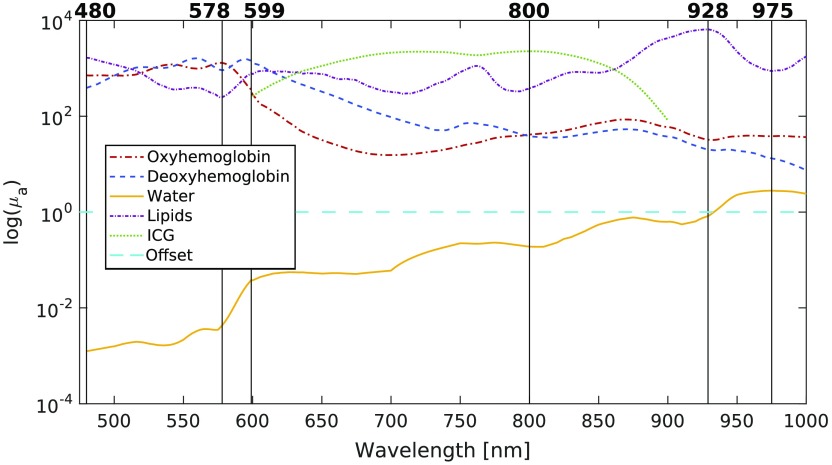
Wavelengths chosen for decoupling ${\mathrm{HbO}}_{2}$, HbR, $H_{2}O$, ICG, lipids, and a linear offset.

Depending on the tissue being imaged, more (or different) absorbers may need to be considered. They can easily be included in the selection process by adding their associated spectra to the matrix of pathlength-modulated bases ($\mu_{a}L$) used to compute the singular values. As a demonstration, the spectra of ICG (for a concentration of 1290  μM), lipids, and a linear offset (which removes linear background sources) were added to the basis spectra matrix, and 6 wavelengths were selected with these additional components. As in the 3 wavelength selection case, optical properties and concentrations were chosen randomly 10 times within the ranges outlined in Table 1, for concentrations of ICG and lipids varying between 0.02 and 0.06 (%). The optimal wavelengths found by our algorithm are presented in Fig. 5. The average RMS error of the concentration estimates generated with these wavelengths was compared with the average errors generated with other algorithms, and the results are summarized in Table 2.

**Table 2:** Average RMS error for fitting six components.

Selection method	Linear spacing	Random selection	Condition number	Smallest singular value	Product of singular value
Average RMS error	0.39	1.45	0.37	0.30	0.13

Once again, selecting wavelengths based on the product of singular values yielded the smallest RMS error and outperformed previously published algorithms.

## 4 Practical Application

**Fig. 6:**
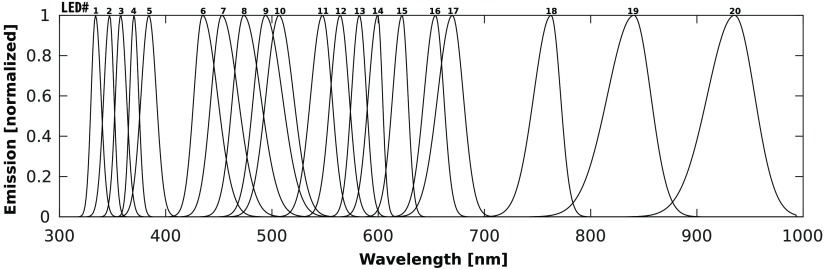
Emission spectra of 20 LEDs considered for diffuse reflectance acquisition of ${\mathrm{HbO}}_{2}$, HbR, and $H_{2}O$.

The selection algorithm outlined in this paper is easily adapted to multiple practical situations. As an example, it is used to select the 3 best LEDs for fitting ${\mathrm{HbO}}_{2}$, HbR, and $H_{2}O$ from an inventory of 20 possibilities. The input set of LEDs was taken from Gareau.^11^ Although LED emission spectra usually have varying amplitudes, they have been normalized for this analysis to account only for their emission wavelength. The normalized spectra are shown in Fig. 6.

LEDs were selected by the algorithm outlined in this paper, by replacing the $\mu_{a}L$ matrix with the following LED matrix

$$[\begin{array}{ccc} \int \mu_{a,\mathrm{oxy}}L\;{\mathrm{LED}}_{1}d\lambda & \int \mu_{a,\mathrm{deoxy}}L\;{\mathrm{LED}}_{1}d\lambda & \int \mu_{a,\mathrm{water}}L\;{\mathrm{LED}}_{1}d\lambda \\ \int \mu_{a,\mathrm{oxy}}L\cdot {\mathrm{LED}}_{2}d\lambda & \int \mu_{a,\mathrm{deoxy}}L\;{\mathrm{LED}}_{2}d\lambda & \int \mu_{a,\mathrm{water}}L\;{\mathrm{LED}}_{2}d\lambda \\ \vdots & \vdots & \vdots \\ \int \mu_{a,\mathrm{oxy}}L\;{\mathrm{LED}}_{20}d\lambda & \int \mu_{a,\mathrm{deoxy}}L\;{\mathrm{LED}}_{20}d\lambda & \int \mu_{a,\mathrm{water}}L\;{\mathrm{LED}}_{20}d\lambda \end{array}],$$ (6)

where ${\mathrm{LED}}_{i}$ is the emission spectrum of LED i. This matrix considers the bandwidth of the LED’s emission as well as the modulated absorption spectra of the components fitted.

Executing the selection algorithm on this matrix returned the 3 best LEDs: LED# 9, LED# 17, and LED# 20 centered at 496, 668, and 935 nm, respectively, and yielded wavelength peaks having slight variations from the optimal wavelengths picked by our algorithm. This outcome is explained by the fact that, when dealing with LEDs, not all wavelengths are available and that other factors, such as LED bandwidth, are considered.

## 5 Conclusion

This paper presented and evaluated a wavelength selection criterion that produced improved results relative to previously published strategies by maximizing the product of singular values of the scattering-modulated absorption matrix. Using this approach, three optimal wavelengths were found for diffuse reflectance spectral fitting of ${\mathrm{HbO}}_{2}$, HbR, and $H_{2}O$. These optimal wavelengths proved to be stable over a wide range of optical absorption and scattering properties. The method is easily extended to more and different absorbers and achieved similarly improved results. Modifications to the algorithm were demonstrated to account for LED broadband emission spectra instead of distinct discrete wavelengths, and the process was used to select LEDs that are optimal for fitting blood components. While this paper prioritized the diffuse reflectance fitting of blood, the algorithm is readily applied to applications that require spectral fitting of other absorbers and/or fluorophores.
